# Modified Gold Nanoparticles for Efficient Delivery of Betulinic Acid to Cancer Cell Mitochondria

**DOI:** 10.3390/ijms22105072

**Published:** 2021-05-11

**Authors:** Olakunle Oladimeji, Jude Akinyelu, Aliscia Daniels, Moganavelli Singh

**Affiliations:** Nano-Gene and Drug Delivery Group, Discipline of Biochemistry, University of KwaZulu-Natal, Private Bag X54001, Durban 4000, South Africa; kunle1433@gmail.com (O.O.); jude.akinyelu@fuoye.edu.ng (J.A.); DanielsA@ukzn.ac.za (A.D.)

**Keywords:** mitochondrial targeting, gold nanoparticles, cancer chemotherapy, drug delivery, betulinic acid, laminin receptor

## Abstract

Advances in nanomedicine have seen the adaptation of nanoparticles (NPs) for subcellular delivery for enhanced therapeutic impact and reduced side effects. The pivotal role of the mitochondria in apoptosis and their potential as a target in cancers enables selective induction of cancer cell death. In this study, we examined the mitochondrial targeted delivery of betulinic acid (BA) by the mitochondriotropic TPP^+^-functionalized epigallocatechin gallate (EGCG)-capped gold NPs (AuNPs), comparing the impact of polyethylene glycol (PEG) and poly-L-lysine-graft-polyethylene glycol (PLL-g-PEG) copolymer on delivery efficacy. This included the assessment of their cellular uptake, mitochondrial localization and efficacy as therapeutic delivery platforms for BA in the human Caco-2, HeLa and MCF-7 cancer cell lines. These mitochondrial-targeted nanocomplexes demonstrated significant inhibition of cancer cell growth, with targeted nanocomplexes recording IC_50_ values in the range of 3.12–13.2 µM compared to that of the free BA (9.74–36.31 µM) in vitro, demonstrating the merit of mitochondrial targeting. Their mechanisms of action implicated high amplitude mitochondrial depolarization, caspases 3/7 activation, with an associated arrest at the G0/G1 phase of the cell cycle. This nano-delivery system is a potentially viable platform for mitochondrial-targeted delivery of BA and highlights mitochondrial targeting as an option in cancer therapy.

## 1. Introduction

The continuous rise in cancer-associated mortality against a backdrop of advances in drug discovery highlights the prevailing challenges of existing therapeutic interventions for cancer. Cancer remains the second leading cause of death worldwide, with approximately 9.6 million deaths recorded in 2018 and another 18 million new cases reported in the same year [1]. While traditional treatment approaches such as surgery and radiotherapy have their limitations, the major demerits of chemotherapy are poor bioavailability, a low therapeutic index, and a marked increase in normal cell cytotoxicity on any compensatory dose increase [2].

The idea of a “magic bullet” in the treatment of diseases proposed by Paul Ehrlich, suggesting the precise delivery of therapeutics to their active sites may have inspired the targeted delivery concept, which has gained traction in recent times [3]. In cancer therapy, the application of this concept has seen the development of nano-delivery systems engineered to target and accumulate in cancerous tissues. Their application significantly enhances drug pharmacokinetics, minimizes side effects, and improves the drug’s therapeutic index [2,4]. Further advances in this research area have also led to the introduction of optimized systems suitable for efficient delivery of the cargo to molecular targets localized in intracellular organelles [5]. Recent reports revealed that nanoformulations targeted to organelles such as the mitochondria, endosome, lysosome, nucleus, ribosome and the Golgi apparatus, demonstrated efficacy in the delivery of genes, proteins and drugs, thus highlighting their potential application in clinical scenarios [6,7]. It is anticipated that the preferential targeting of subcellular compartments with nanomedicines would significantly improve treatment selectivity and biocompatibility, with the potential of achieving higher therapeutic indices at lower doses. The central role of mitochondria in bioenergetics and apoptosis signaling has made them a focus of clinical research, and a therapeutic target for diseases in recent years. Although apoptosis is a well-regulated physiological process, certain extracellular agents have been shown to modulate mitochondria function and induce apoptosis, thus presenting an in-road to preferentially targeting the organelle in related disease conditions [8].

Betulinic acid (BA) is a pentacyclic triterpenoid, from a subclass of terpenes composed of isopentenyl pyrophosphate oligomers, that have been studied for their medicinal potential [9,10]. BA demonstrates potent activity against bacterial and viral infections, inflammation, malaria, and cancer [10,11]. Research has shown that the anticancer effect of BA is linked to its impact on the mitochondria. BA induces membrane permeability transition (MPT) in cancer mitochondria, consequently triggering the dissipation of mitochondrial membrane potential, cytochrome c release, caspase activation and finally, apoptosis [12,13,14]. A major drawback to BA’s clinical potential is its poor solubility, which, coupled with other common limitations such as high blood clearance and low specificity, impacts its efficacy in vivo. To improve the therapeutic effectiveness of the drug on the cancer cell mitochondria, a nano-delivery system with the capacity to successfully navigate physiological and cellular barriers and ensure the delivery of the payload to the mitochondria for maximum impact is imperative [15].

Biocompatibility is a critical factor in the efficacy of nano-delivery platforms. The green or organic synthesis of inorganic nanoparticles (NPs) offers, among other things, the elimination of toxicities associated with chemical synthesis, as well as the transfer of bioactive properties from plant-based reductants. Green synthesis of inorganic NPs employs the reductive power of bio-extracts to affect the reduction of metallic salts from their initial oxidative state to zero [16]. Generally, green synthesized NPs adopt the unique features of bio-extracts employed in their synthesis. Organic synthesis with extracts from fungi, algae, plants, and bacteria have given rise to NPs with inherent antioxidant, antibacterial and self-targeting properties. EGCG has been reported to show an affinity for the 67 kD laminin receptor (67LR), that is overexpressed in most aggressive tumors [17,18,19,20]. The laminin receptor-dependent uptake of EGCG-reduced radioactive gold NPs (AuNPs) by cancer cells has been reported [21]. Considering the potential inherent in the preferential targeting of therapeutics to intracellular sites of action to bring about improved bioavailability and therapeutic efficacy at a lower dose with tolerable side effects, we have studied the capacity of EGCG-capped, laminin receptor-avid AuNPs for mitochondrial-targeted delivery of BA in selected cancer cells in vitro. The decoration of an inorganic NP surface with biodegradable polymers has been reported to improve NP biocompatibility, stability, and influence cargo release [22]. PLL, a biocompatible and biodegradable polymer of the amino acid L-lysine exerts these effects, and apart from the provision of functional amino groups for the easy conjugation of other components, its net electropositive charge promotes an electrostatic interaction with the electronegative cellular membrane, aiding NP uptake [23,24]. The pegylation of NPs, among other things, confers stealth properties and correlates with increased residence time in vivo [22,25]. Therefore, we assessed the impact of surface design on NP efficiency, comparing the activities of PEG-coated nanocomplexes to the PLL-g-PEG-coated variant. To the best of our knowledge, this study is the first attempt at preferentially targeting BA to the mitochondria.

## 2. Results

### 2.1. Synthesis and Functionalization of EGCG-Capped AuNPs

**Figure 1:**
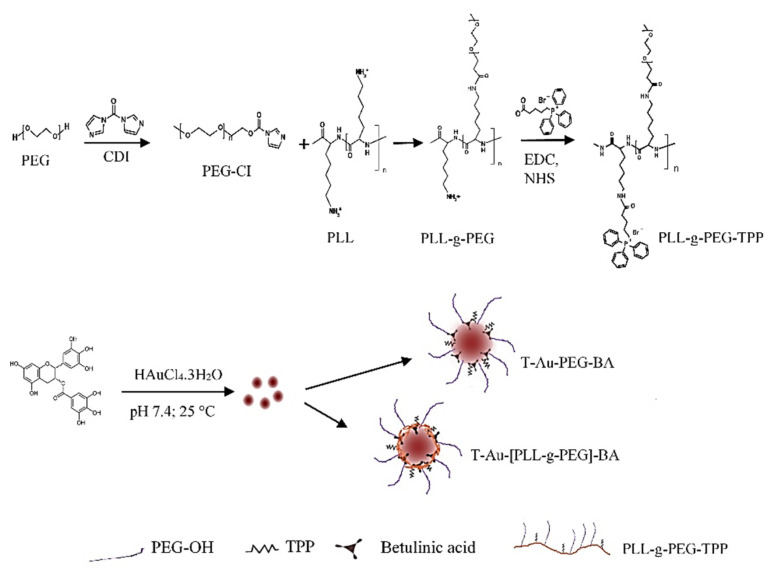
The synthesis of EGCG-capped NPs, and the engineering of the targeted T-Au-PEG-BA and T-Au-[PLL-g-PEG]-BA nanoparticles.

EGCG-capped AuNPs were synthesized by the reduction of gold (III) chloride with the green tea polyphenol, EGCG, as illustrated in Figure 1. Surface functionalization of EGCG-capped NPs involved coating with either PEG, or a graft polymer of PLL-PEG (PLL-g-PEG) to produce two polymer-coated variants, the Au-PEG and the Au-[PLL-g-PEG] NPs, respectively. Further functionalization included the conjugation of the drug BA, and the lipophilic moiety, triphenylphosphine (TPP^+^) for enhanced mitochondria localization.

### 2.2. UV-Vis Spectroscopy

**Figure 2:**
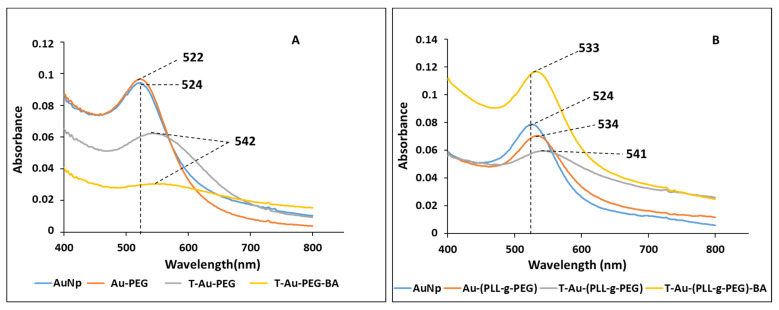
UV-vis of nanoparticles and drug nanocomplexes showing changes in surface plasmon resonance: (**A**) PEG functionalized AuNPs; (**B**) PLL-g-PEG coated AuNPs.

The synthesis and functionalization of EGCG-capped AuNPs were monitored by UV-vis spectroscopy. Inorganic NPs generally show distinct optical properties, which are representative of their physical features such as size and stability [26,27]. The successful functionalization of a NP results in changes in the oscillation state of its surface electrons, otherwise referred to as localized surface plasmon resonance (SPR), manifesting as change in optical properties. AuNPs generally show a strong absorbance peak between 500 and 600 nm, and in this study, EGCG capped AuNPs had a strong absorbance peak at 524 nm, comparable to citrate capped AuNPs (520 nm) [28,29]. Subsequent surface designs resulted in band shifts and reduction in peak intensities in Au-PEG (522 nm), T-Au-PEG and T-Au-PEG-BA (542 nm), with the blueshift on PEG conjugation indicating a reduction in size, the redshift to 542 nm on TPP^+^ and BA showing a slight reduction in NP stability compared to unfunctionalized AuNPs, with the widening of the peak indicating an increase in dispersity. Similarly, the coating with PLL-g-PEG resulted in a band shift to 534 nm, while the conjugation of TPP+ and BA resulted in a shift to 541 and 533 nm, respectively (Figure 2), indicating an increase in size or slight reduction in stability compared to the unfunctionalized AuNPs. Overall, the PLL-g-PEG coated NPs seemed to be the more stable of the two designs. The band shifts and reduction in peak intensity are suggestive of a successful binding of the polymer and drug.

### 2.3. Fourier-Transform Infrared Spectroscopy (FTIR)

**Figure 3:**
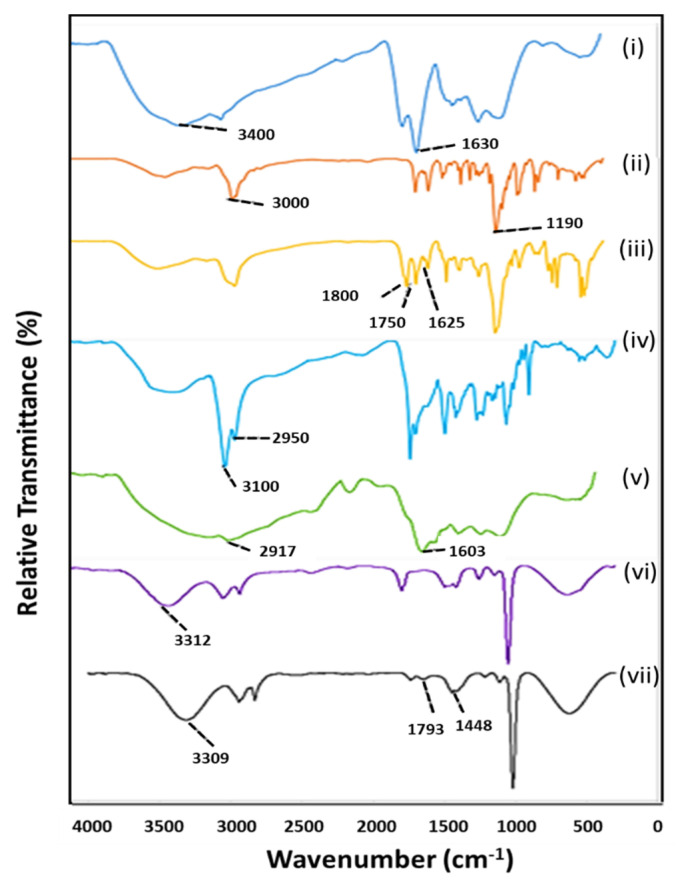
FTIR of nanoparticles showing changes in bond vibrations of respective functional groups. (**i**) AuNP, (**ii**) Au-PEG, (**iii**) T-Au-PEG, (**iv**) T-Au-PEG-BA, (**v**) Au-[PLL-g-PEG], (**vi**) T-Au-[PLL-g-PEG], (**vii**) T-Au-[PLL-g-PEG]-BA.

The frequency of vibration of molecules upon absorption of electrochemical radiation is distinctive for individual molecules. These vibrations, represented as band shifts at characteristic wavelengths in the IR spectrum are applied in the analysis of organic compounds, especially in the identification of functional groups [30]. The synthesis and subsequent functionalization of NPs with polymers, drug, and the targeting moiety were monitored and confirmed by FTIR analysis. As represented in Figure 3, EGCG capped AuNPs showed stretching vibrations of O-H (3400 cm^−1^) and C-H (3000 cm^−1^) of the aromatic rings characteristic of the polyphenol groups of EGCG. In addition, the aromatic skeletal vibrations at 1630 cm^−1^ also of the EGCG moiety further confirmed the successful capping of AuNPs by the polyphenol. The successful functionalization of AuNPs with PEG was evident from the stronger peak at 3000 and 1190 cm^−1^ due to the stretching vibrations of the C-H bonds and C-O bonds of the polymer, respectively.

The lipophilic cation TPP+ moiety of the targeted NP (T-Au-PEG) coupled by Steglich esterification to the OH groups of Au-PEG, showed characteristic skeletal vibrations of its three aromatic rings at 1800, 1750 and 1625 cm^−1^. Similarly, the very strong vibrations of the C=O group at 1681 cm^−1^, and those of the CH_3_, CH_2_, and CH, at 2950 and 3000 cm^−1^ in T-Au-PEG-BA, confirmed the loading of BA into the NPs [31]. The coating of NPs with PLL-g-PEG to yield the Au-PLL-g-PEG, showed the characteristic stretch vibration for the repeating CH_2_ groups of PLL at 2917 cm^−1^, and the secondary amine groups (NH) at 1603 cm^−1^. The subsequent conjugation of TPP^+^ is evident from the stretching vibrations of the C-H bonds of the benzene rings at 3312 cm^−1^, and the stretching vibration of CH_2_ and CH_3_ groups at 2945 and 2835 cm^−1^, respectively. The loading of BA in T-Au-[PLL-g-PEG]-BA was confirmed by the amplification of IR peaks at 3309 cm^−1^ for OH stretching vibration, and at 2943 cm^−1^ for CH_3_, CH_2_ and CH groups. Furthermore, the bands at 1793 cm^−1^ (shoulder) and 1648 cm^−1^ are representative of the C=O group of BA, while the signal at 1448 cm^−1^ is due to the bending vibrations of the CH_3_ and CH_2_ groups of the aliphatic ring of the drug [30].

### 2.4. Drug Loading, TEM and NTA

Au-PEG-BA and T-Au-PEG-BA had loading efficiencies of ~25.4%, while Au-PLL-g-PEG-BA and T-Au-PLL-g-PEG-BA had loading efficiencies of ~21% (Table 1). Table 1 also reflects the NTA hydrodynamic size, zeta potential and polydispersity indices of all NPs.

**Figure 4:**
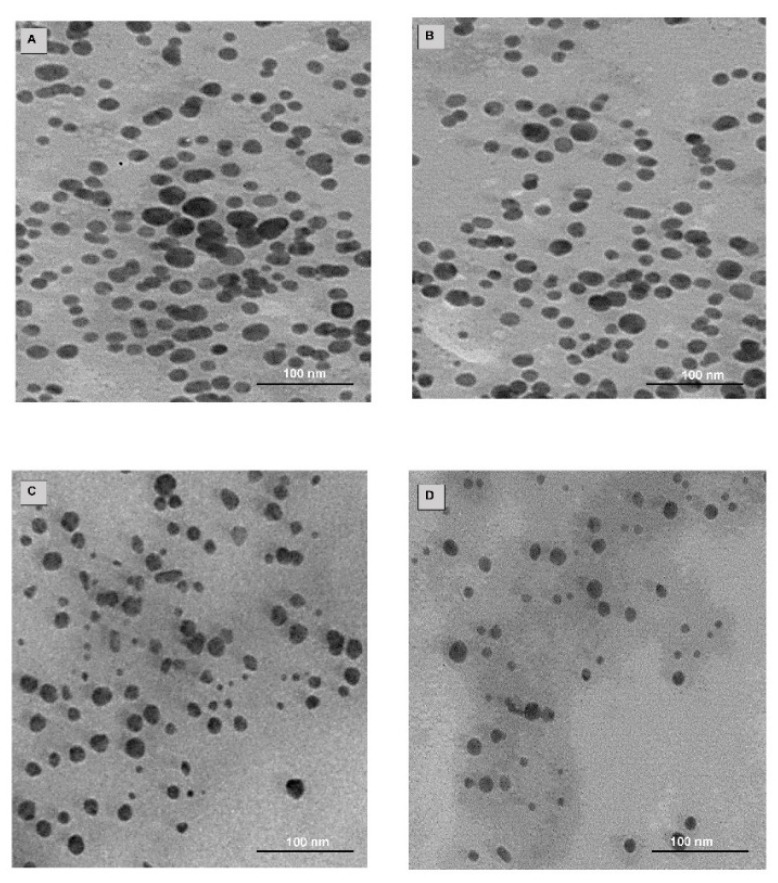
TEM images of nanoparticles: (**A**) AuNPs, (**B**) AuNP-BA, (**C**) T-Au-PEG-BA, (**D**) T-Au-[PLL-g-PEG]-BA.

TEM showed stable, spherical NPs (Figure 4) with an average size diameter of 15 nm. Analysis following surface design showed negligible change in particle size, and no agglomeration. The hydrodynamic diameter (NTA) for AuNPs was in the 127 nm range, while subsequent modification resulted in a reduction in the diameter to 110.1 and 97.1 nm for Au-PEG-BA and T-Au-PEG-BA, respectively. Upon coating with PLL-g-PEG and TPP^+^-PLL-g-PEG, an increase in NP diameter to 147.2 nm and a reduction to 119 nm were recorded, respectively (Table 1). The polydispersity index (PDI) provides information on the size distribution of NPs. Generally, NPs with low PDI values are monodispersed and uniform in size [32]. The PDI estimations for the nanocomplexes studied suggests moderate dispersity with values closer to 0.1, except for the Au-PEG-BA nano-construct with a PDI value of 0.22, suggesting some polydispersity.

The zeta potential measurement which informs on the surface charge of the NP with respect to that of the conducting fluid, in this case water (pH 7), is a marker for NP stability and biomolecular interactions. The PEG functionalization of the AuNPs only effected marginal changes, with the zeta potential remaining negative even upon further functionalization with TPP^+^. Conversely, coating with the cationic PLL-g-PEG and TPP^+^-PLL-g-PEG resulted in positive surface charge for the NPs, of 11.8 and 23.4 mV, respectively. Overall, the zeta potentials recorded indicated that the nanocomplexes were stable, however, the negative charge of T-Au-PEG-BA and Au-PEG-BA could have adversely influenced their interaction with the cell and mitochondria due to the negative potentials (Ψ) of both membranes.

### 2.5. Cellular Uptake and Mitochondrial Targeting

**Figure 5:**
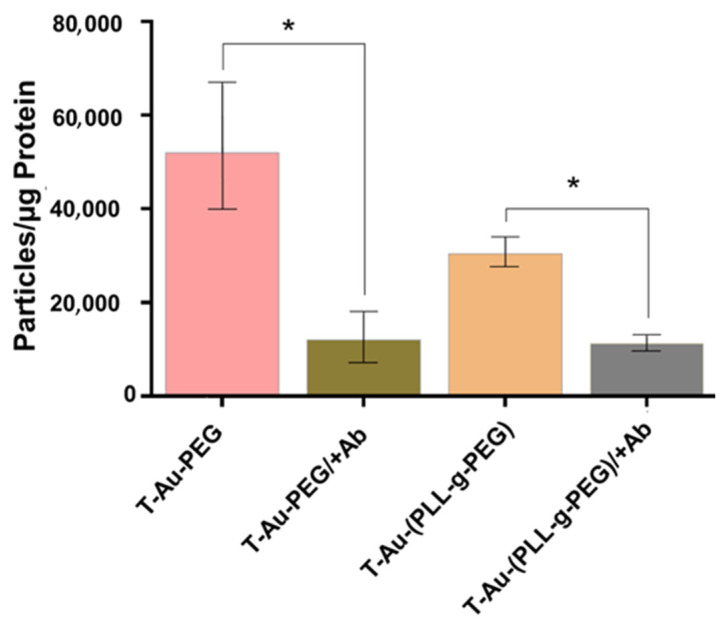
Graphical representation of the laminin dependent uptake of targeted nanoparticles in HeLa cells. Data is represented as mean ± SD (*n* = 3). (* *p* < 0.05).

The ability of NPs to cross the cell membrane bilayer and translocate efficiently to subcellular targets is pivotal to their application in therapeutic delivery. Given earlier reports on the affinity of EGCG for the 67-kDa laminin receptor [21,33], the laminin receptor dependent uptake of T-Au-PEG and T-Au-PLL-g-PEG was studied in the HeLa cell line due to its high expression of the receptor [34]. Cellular uptake in cells preincubated with the laminin receptor antibody were generally lower compared to cells not treated with the antibody (Figure 5). For T-Au-PEG NPs, uptake was significantly lower in preincubated cells (T-Au-PEG: 51 980.98 particles/µg protein; T-Au-PEG/+Ab: 12,059.37 particles/µg protein), thus showing a strong dependence on laminin receptor mediated uptake. Although recording a lower level of uptake compared to T-Au-PEG, a statistically significant reduction in uptake upon receptor blocking was also recorded for the T-Au-[PLL-g-PEG] NPs with 30,451.87 particles/µg protein, and T-Au-[PLL-g-PEG]/+Ab with 11,273.19 particles/µg protein). The lower levels observed for T-Au-[PLL-g-PEG] NPs can be attributed to their larger size which may have an impact on the efficiency of NP uptake.

**Figure 6:**
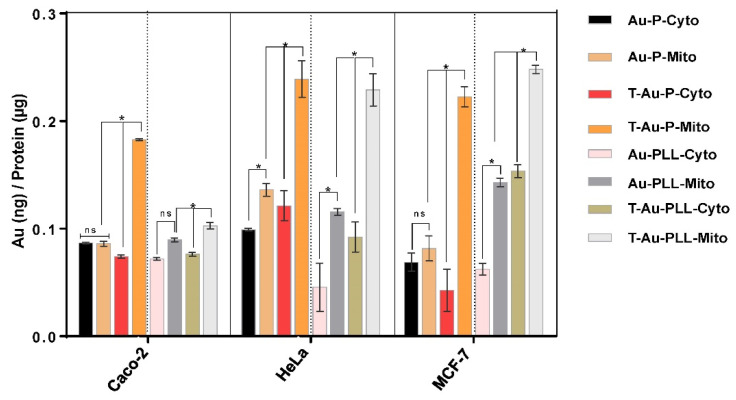
Cytosolic and mitochondrial distribution of mitochondrial targeted and nontargeted nanoparticles as determined by ICP-OES. Data is represented as mean ± SD (*n* = 3); * *p* < 0.05; ns = no significant difference.

The distribution of NPs between the cytoplasm and mitochondria, in the Caco-2, MCF-7 and HeLa cell lines, was determined after a 12 h period by the differential centrifugation of the cell lysate to obtain the cytoplasmic and mitochondrial fractions, and thereafter analyzed by ICP-OES to obtain a quantitative estimate of the mitochondrial localization efficiency of these NPs. Significant mitochondrial localization was achieved by T-Au-PEG and T-Au-[PLL-g-PEG] NPs compared to their untargeted counterparts (Au-PEG and Au-[PLL-g-PEG]) (Figure 6).

Furthermore, in all three cell lines, mitochondrial localization was higher for T-Au-[PLL-g-PEG] NPs compared to T-Au-PEG NPs. Interestingly, mitochondrial localization for the two targeted NPs was highest in the MCF-7 and lowest in Caco-2 cells. While the presented data does not establish the rate of NP uptake in the different cell lines, it suggests that the intracellular trafficking of NPs may vary for the respective cell lines.

### 2.6. Cytotoxicity

**Figure 7:**
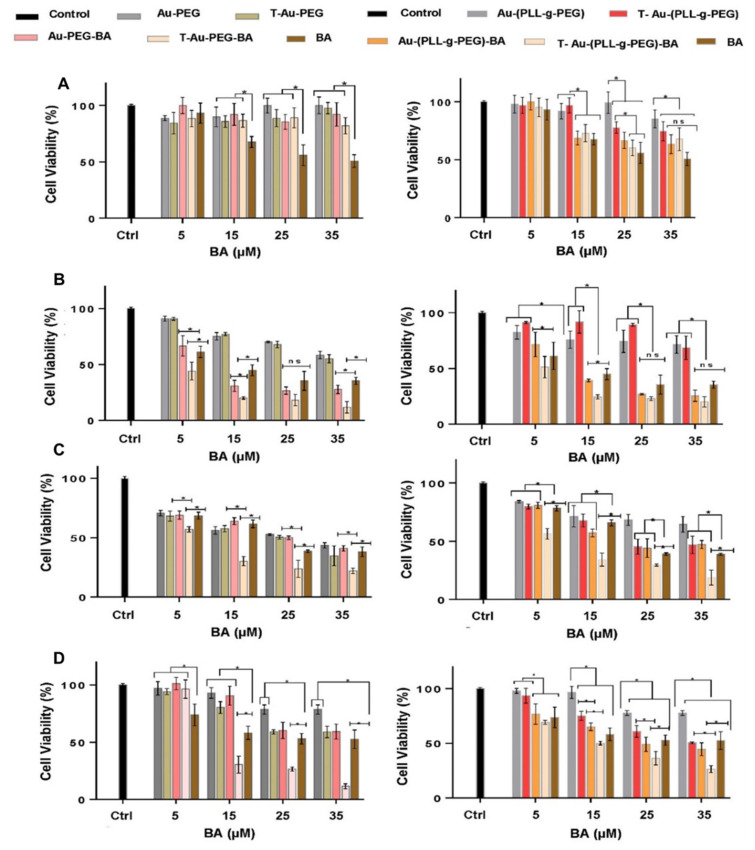
Effect of nanoparticles and drug nanocomplexes on cell viability in (**A**) HEK 293, (**B**) Caco-2, (**C**) HeLa and (**D**) MCF-7 cells. Au-PEG-BA; Au-[PLL-g-PEG]-BA = untargeted nanocomplexes, T-Au-PEG-BA; T-Au-[PLL-g-PEG]-BA = targeted nanocomplexes, and BA= free drug. Data is represented as mean ± SD (*n* = 3); * *p* < 0.05); ns = no significant difference.

The impact of targeted and untargeted nanocomplexes, and the free drug on cell proliferation was assessed in the HEK293, Caco-2, HeLa and MCF-7 cells, using the colorimetric MTT assay (Figure 7).

The drug-loaded nanocomplexes tested were reported in the concentrations of their respective BA content (µM) for easy comparison with free BA, while the corresponding volume of drug-loaded NPs used for each drug concentration was adopted for drug free nanocomplexes. Negligible cytotoxicity was recorded for all NPs in the HEK293 cells, suggesting that these normal cells showed good tolerance to these treatments. Similarly, drug loaded NPs showed low cytotoxicity, while free BA recorded marked cytotoxicity in normal HEK293 cells, recording an IC_50_ value of 32.4 µM compared to >80 µM recorded for T-Au-PEG-BA and T-Au[PLL-g-PEG]-BA. The efficacy of the NPs in mitochondrial accumulation, and subsequent delivery of their cargo is evident from the low IC_50_ values recorded for targeted nanocomplexes compared to their nontargeted counterparts and the free drug. As presented in Table 2, the estimated IC_50_ values for T-Au-PEG-BA in Caco-2, HeLa and MCF-7 cells are 3.13, 6.51 and 13.2 µM, respectively, which are markedly low compared to the 8.20, 25.37 and 53.74 µM recorded for Au-PEG-BA; and 9.74, 17.73 and 36.31 µM recorded for free drug. Similarly, T-Au[PLL-g-PEG]-BA inhibited proliferation at levels significantly higher compared to Au[PLL-g-PEG]-BA and BA, recording low IC_50_ values in Caco-2, HeLa and MCF-7 cells (3.12, 3.26 and 13.13 µM, respectively), compared to the higher IC_50_ values recorded by Au[PLL-g-PEG]-BA (5.72, 23.64 and 22.25 µM) and free BA (9.74, 17.73, 36.31 µM) in the same cells. In spite of the lower mitochondrial accumulation seen in T-Au-PEG-BA (Figure 6), its impact on cellular proliferation is not different from that of T-Au[PLL-g-PEG]-BA, which suggests the possibility of extra-mitochondrial targets for T-Au-PEG-BA.

### 2.7. Effect on Mitochondrial Membrane Potential

**Figure 8:**
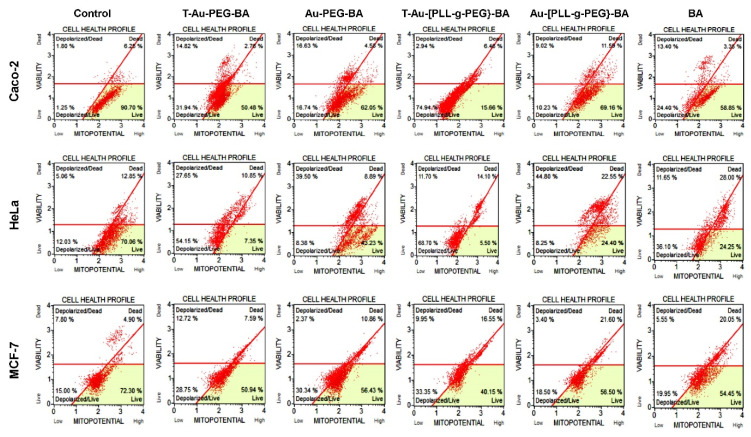
Cytographs of the effect of the targeted and untargeted nanocomplexes on the mitochondrial membrane potential in Caco-2, HeLa and MCF-7 cells. Au-PEG-BA; Au-[PLL-g-PEG]-BA = untargeted nanocomplexes, T-Au-PEG-BA; T-Au-[PLL-g-PEG]-BA = targeted nanocomplexes, and BA = free drug. Top left quadrat: cell death by mitochondrial depolarization; top right quadrat: non-mitochondria dependent cell death; lower left quadrat: mitochondrial depolarization in live cells; lower right quadrat: live cells.

**Figure 9:**
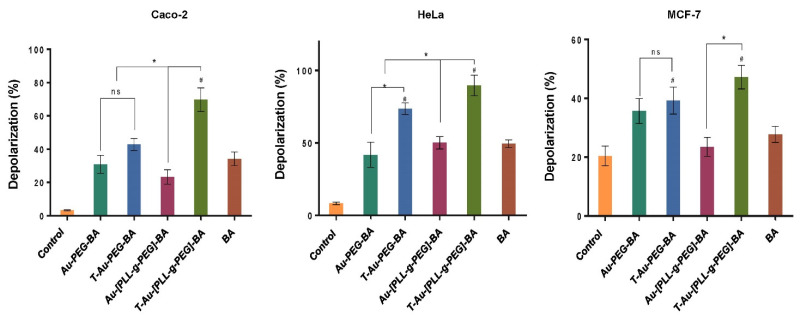
Graphical representation of the effect of targeted and untargeted nanocomplexes on the mitochondrial membrane potential in Caco-2, HeLa and MCF-7 cells. Data is represented as mean ± SD (*n* = 3). * vs targeted or untargeted nanocomplex, # vs. free drug at *p* < 0.05). ns = no significant difference.

To elucidate the impact of targeted nanocomplexes on the mitochondria and their underlying mechanism of action, the effects of all nanocomplexes and free drug on the mitochondria in the three cancer cell lines was investigated. Due to their efficient mitochondrial localization, the targeted nanocomplexes, T-Au-PEG-BA and T-Au-[PLL-g-PEG]-BA showed significant impact on mitochondrial membrane potential compared to the untargeted and free BA (Figure 8 and Figure 9).

Results suggest a significant impact by the cationic T-Au-[PLL-g-PEG]-BA recording 74.94%, 68.7% and 33.35% depolarization in the Caco-2, HeLa and MCF-7 cell lines, compared to the 31.94%, 54.15% and 28.75% observed for T-Au-PEG-BA. Furthermore, the activity of the nanocomplexes in the MCF-7 cells was generally lower in comparison with their activity in the other cells, highlighting the selective sensitivity of BA to certain cancers and confirming previous reports [10,35].

### 2.8. Impact on Caspases 3 and 7 Activity

**Figure 10:**
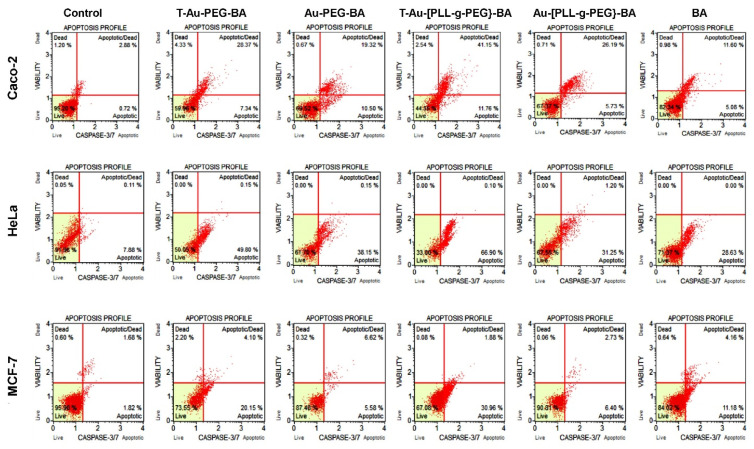
Cytographs showing the effect of targeted and untargeted nanocomplexes on caspases 3 and 7 in Caco-2, HeLa and MCF-7 cells. Au-PEG-BA; Au-[PLL-g-PEG]-BA = untargeted n nanocomplexes, T-Au-PEG-BA; T-Au-[PLL-g-PEG]-BA = targeted nanocomplexes, and BA = free drug. Top left quadrat: cell death by non-apoptotic mechanisms; top right quadrat: cell death by caspase dependent apoptosis; lower left quadrat: Live cells; lower right quadrat: cells undergoing caspase dependent apoptosis.

**Figure 11:**
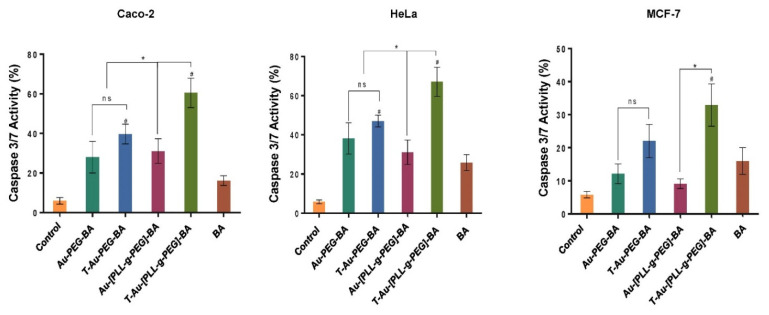
Graphical representation of the effect of targeted and untargeted nanocomplexes on caspases 3 and 7 in Caco-2, HeLa and MCF-7 cells. Data is represented as mean ± SD (*n* = 3). * vs. targeted or untargeted nanocomplex, # vs. free drug at *p* < 0.05. ns = no significant difference.

The activity of caspases 3 and 7 (Figure 10 and Figure 11) were assessed as markers of apoptosis initiation, using a fluorescent substrate—DEVD of the executioner proteases, and the cell death marker 7-AAD. From Figure 10 and Figure 11 it can be observed that the highest levels of activation were recorded by T-Au-[PLL-g-PEG]-BA in all three cells, with a total of 52.81%, 67% and 32.84% apoptotic cell populations compared to the 35.71%, 49.95% and 24.25% for T-Au-PEG-BA, which is in tandem with results from the mitochondrial membrane potential dissipation. Apart from the demonstrated efficacy of the targeted nanocomplexes, they also demonstrated appreciable improvement over free BA in caspase induction.

### 2.9. The Effect of Targeted Nanocomplexes on Apoptosis Induction

**Figure 12:**
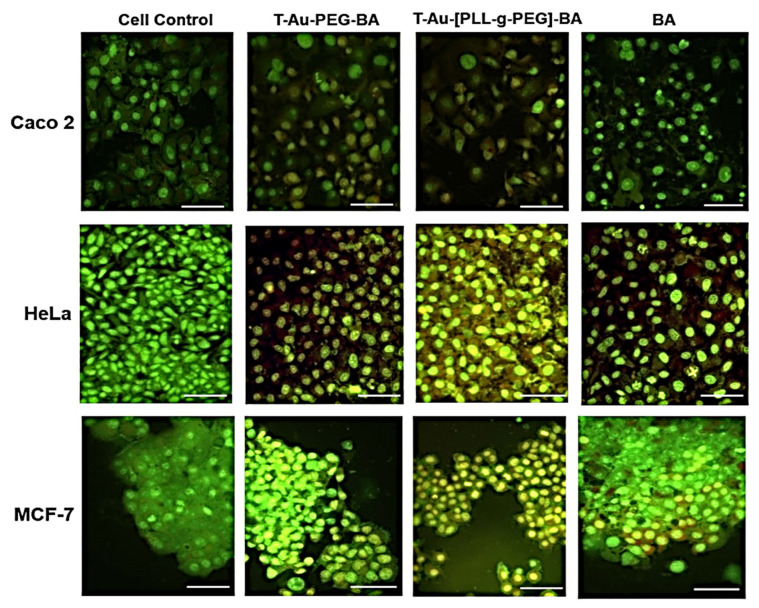
Sectional fluorescent images of acridine orange/ethidium bromide stained cells showing apoptosis induction by targeted nanocomplexes. T-Au-PEG-BA; T-Au-[PLL-g-PEG]-BA = targeted nanocomplexes, and BA = free drug. Untreated cells and BA served as controls. Scale bar = 100 µm.

Following incubation with the nanocomplexes and drug at predetermined concentrations, cells were stained with the AO/EB dual stain [28], to determine the extent of apoptosis in vitro. Fluorescent images showing cytomorphological changes indicative of apoptosis were noticed for treated cancer cell lines at varying degrees (Figure 12).

In comparison to the control cells (nontreated), morphological changes such as cell shrinkage and membrane blebbing indicative of apoptosis were observed in treated cells. These changes were moderate in BA treated cells, but generally severe in cells exposed to nanocomplexes. The dual staining technique is able to distinguish between early apoptotic cells, from cells at the final stage of apoptosis, with light yellow fluorescence indicating early apoptosis, and orange fluorescence suggesting cells are in the late stage of apoptosis. Overall, both targeted nanocomplexes showed significant improvement over free BA, demonstrating the importance of these NPs to enhance drug bioavailability and improve pharmacokinetics.

### 2.10. The Impact of Targeted Nanocomplexes on Cell Cycle Progression

**Figure 13:**
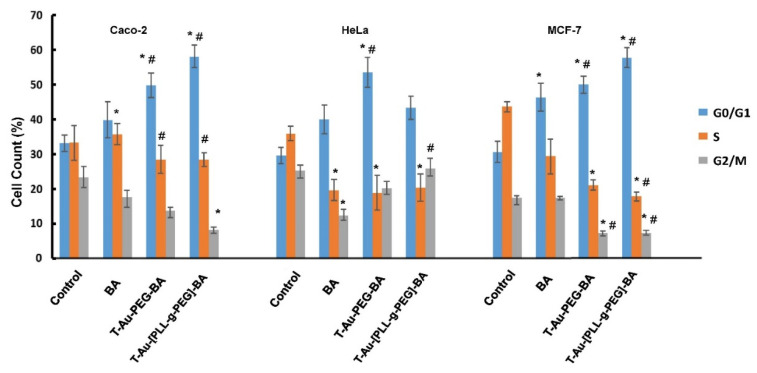
Graphical representation showing the effect of T-Au-PEG-BA, T-Au-[PLL-g-PEG]-BA and BA on cell cycle progression. T-Au-PEG-BA; T-Au-[PLL-g-PEG]-BA = targeted nanocomplexes, and BA= free drug. Data is represented as mean ± SD (*n* = 3). * vs. control, # vs. free drug at * *p* < 0.05.

The targeted nanocomplexes were then studied further to determine their impact on the cell cycle. Cells were incubated with an equimolar concentration of the drug and the nanocomplexes were assessed for distribution across the G0/G1, S and G2/M phases. There was appreciable cell distribution across the S and G2/M phases in the control group for the Caco-2, HeLa and MCF-7 cells, compared to the treated groups (Figure 13), which is indicative of the high rate of proliferation in this group of cells.

Upon addition of the various treatments, a comparable decrease in cell population for the two phases was noticed, with a corresponding increase in cell number at the G0/G1 phase. In the Caco-2 cells, a significant increase in the number of cells arrested at the G0/G1 phase was observed for T-Au-[PLL-g-PEG], recording a 1.8-fold increase over the control. T-Au-PEG-BA and BA also recorded increases. In the MCF-7 cells, the trend was similar, with the G0/G1 cell population increasing from 30% in the control to 46%, 57%, and 66% in BA, T-Au-PEG-BA, and T-Au-[PLL-g-PEG], respectively. However, in the HeLa cells, the highest G0/G1 arrest was observed for T-Au-PEG-BA, recording a 1.83-fold increase compared to that for T-Au-[PLL-g-PEG] (1.48-fold), further demonstrating the efficacy of the targeted nanocomplexes over the free drug.

**Figure 14:**
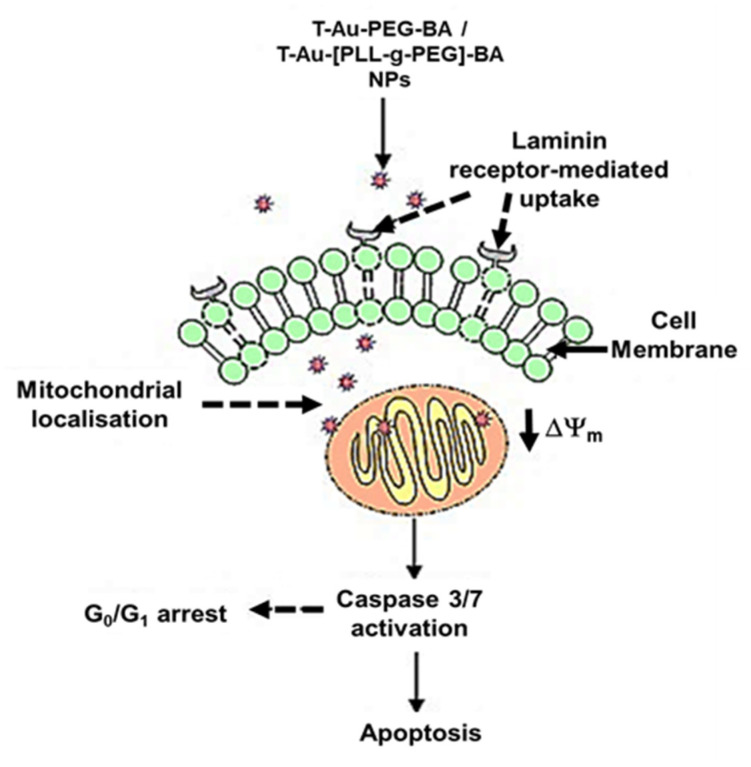
Illustration indicating possible mechanisms involved in cell death.

Figure 14 provides an illustration depicting the cellular uptake, mitochondrial localization and mechanisms involved in cell death.

## 3. Discussion

The identification of the mitochondria as a therapeutic target for cancer chemotherapy initiated the search for therapeutics capable of impacting mitochondrial integrity and inducing apoptosis in cancer cells. Although studies have identified other cytoplasmic targets for BA, the merit for preferentially targeting the mitochondria lies in the high susceptibility of cancer mitochondria to mito-active agents owing to their dysfunctional status, thereby making the approach more specific as the impact on noncancer cells is generally negligible [36,37,38]. Therefore, for efficacy and specificity, the targeted delivery of mitochondrial-active principles has become an emerging area of research.

The organic synthesis of NPs which has seen increasing application in clinical research has shown significantly low environmental toxicity and ease of synthesis. AuNPs were successfully synthesized by exploiting the strong redox properties of the green tea polyphenol, EGCG to reduce the gold chloride. The EGCG-capped AuNPs were further functionalized with PLL and PEG to produce EGCG-capped laminin receptor-avid AuNPs for drug delivery to the mitochondria. The functionalization of NPs with polymers has been reported to improve NP stability, reduce agglomeration, allow for conjugation of bioactive moieties, and importantly, forestall NP immunogenicity while improving stealth properties in vivo [7,39,40]. The covalent attachment of PLL to AuNPs via its amide group has been reported to provide the gold-based nano-delivery system with favorable properties for gene delivery. The many active amino groups of PLL can accept protons at acidic pH and have been observed to significantly promote cell adhesion [41]. The amount of PEG in the formulation was sufficient to promote stabilization and produce NPs and nanocomplexes smaller than 150 nm. Higher densities of PEG may cause the PEG chains to extend further from the NP forming a brush border [42,43]. This may impact on the interaction between the NP and the cells.

The successful synthesis and functionalization of the NPs was confirmed by UV-vis spectroscopy, FTIR and ^1^H NMR (Supplementary Materials Figures S1–S3), with NPs and nanocomplexes exhibiting varying color intensities. The magnitude of absorption and wavelength shifts observed from the UV spectra indicate changes in nanoparticle size and stability upon functionalization, due to its impact on the charge density and oscillation of electrons on NP surface. The presence of the AuNPs was confirmed by an excitation peak at 524 nm, while evidence for functionalization with PEG and PLL, and BA encapsulation were observed as red or blueshifts in the SPR, corresponding to a reduction or increase in NP size, respectively. It has been reported that modification of the surface of AuNPs is often denoted by a significant reduction in the absorption intensity in addition to marginal peak shifts [28], with the broadening of a peak suggesting NP aggregation due to low stability [44]. Upon PEG conjugation, a minimal blueshift in peak wavelength from 524 to 522 nm was observed, reflecting a change in the surface property of the NP, as reported previously [45]. TPP^+^ and BA conjugation resulted in a redshift to 542 nm, indicating a size increase, with the broadening of peaks and reduced intensity suggesting a tendency for agglomeration, especially for the T-Au-PEG-BA nanocomplexes. Although T-Au-PEG-BA had high a zeta potential (Table 2), indicating strong stabilizing repulsive forces between the colloidal particles, a mild to severe agglomeration has been associated with increasing ionic strength of NP suspensions in spite of high zeta potential values. It is suspected that an increase in ionic strength upon addition of TPP^+^ to Au-PEG may have impacted on the stability of T-Au-PEG-BA. In contrast, PLL-g-PEG coated NPs demonstrated significant stability, with the redshifts indicating changes in surface properties and size increase. This observed stability may be a combination of its high zeta potential (Table 1), the steric stabilization conferred by PEG, and the high density of positively charged amines on the NP surface which prevented aggregation. Furthermore, T-Au-[PLL-g-PEG]-BA (533 nm) produced a sharp peak and a blueshift compared to T-Au-[PLL-g-PEG] (541 nm) and Au-[PLL-g-PEG] (534 nm) indicating better stability.

For drug loading, BA was coupled to the NP’s surface by Steglich esterification between the hydroxyl groups on the NP and the carboxylic groups of BA. Although, the functionalized NPs recorded low (<25%) loading efficiency which was lower than that for porous NPs with efficiencies >50%, the values recorded are higher than the 10% benchmark for ideal nanocarriers [46]. It has been suggested that significant therapeutic effects are achievable at lower drug concentrations for subcellular targets such as the mitochondria, with drug release proposed to involve the action of mitochondrial proteases [47]. NP size, surface charge and shape influence their delivery potential, distribution, clearance, accumulation at intended sites via enhanced permeability retention (EPR) and cellular and organellar interactions [48]. TEM revealed small spherical particles which were dark and dense due to the great light scattering properties of gold. As expected, the size of the NPs using TEM were in contrast with that of the hydrodynamic analysis (NTA) which determines the properties of NPs in an aqueous medium which could have resulted in some swelling of the NPs. NTA is a robust technique that provides the size, dispersity and colloidal stability of a NP in real time, by using laser light scattering to track individual particles based on their Brownian motion [2]. The hydrodynamic diameter, also referred to as the Stokes diameter, takes into consideration the presence of a hydration layer around the NP, and is also dependent on the diameter of the NP corona. The initial hydrodynamic diameter of the AuNPs resulted in size changes upon functionalization, which was not very clear from the TEM images. It has been reported that NPs of the sizes <200 nm have good interaction with the cellular membrane and are readily taken up via clathrin-mediated endocytosis [49,50]. All nanocomplexes in this study were favorably under 150 nm in size. Such small sizes are significant in drug delivery, since these drug nanocarriers can easily escape the leaky tumor vasculature to accumulate at the tumor site and induce their anticancer effects [2].

Zeta potential has become a powerful tool for examining the electrostatic forces within the bulk solution and on the surface of the NPs and nanocomplexes [51]. It is de-fined as the magnitude of the electrostatic potential generated on the edge of the slipping plane between the particle and the dispersant medium. The NP interacts with the ions in the dispersant medium [52,53]. The zeta potential is a good measure of NP stability, with values of ±20 mV irrespective of charge being regarded as highly stable. It can also inform on the net surface charge of a NP [54]. When zeta potentials are <−15 or >+15 mV, the attractive forces exceed the repulsive forces resulting in NP aggregation [2]. This was evident for Au-[PLL-g-PEG]-BA which had a low zeta potential (11.8 mV) in addition to being larger (147.4 nm) than the other nanocomplexes. Hence, these nanocomplexes had the tendency to aggregate which could account for the large size recorded. T-Au-PEG and T-Au-[PLL-g-PEG] recorded high zeta potential values of −23.1 and +23.4 mV, respectively, suggesting stability and low tendency for agglomeration. Since NPs with positive zeta potentials are assumed to interact favorably with anionic cell membranes, it is also possible for NPs with negative zeta potential to enter cells and induce their therapeutic activity [53]. The polydispersity index (PDI) provides us with an indication of the uniformity of a particle size in solution. The larger the PDI value, the larger the size distribution in the sample. The PDI value can also provide an insight into particle aggregation, consistency and the efficiency of the modifications on the NP. Generally, monodisperse samples have PDI values less than 0.1 [32], moderately dispersed samples have PDI values between 0.1 and 0.4, and PDI values >0.4 have a broad size distribution [40]. Hence, the formulated NPs and nanocomplexes were close to homogeneity with only Au-PEG-BA being moderately dispersed. Importantly, the functionalization of the AuNPs did not greatly affect their dispersity in solution.

Charged NPs generally are capable of indiscriminate interaction with biomolecules, nevertheless, cationic NPs readily associate with the cellular membrane and enjoy significant cellular uptake by cells compared to anionic NPs. The uptake of NPs into cancer cells is mediated via three classical pathways, the clathrin mediated pathway, caveolae dependent endocytosis and macropinocytosis. However, the route of uptake is determined by the size, shape and surface charge of the NPs. Cellular targeting using receptor agonists as the homing moiety for NPs help improve uptake, allows for cell-specific delivery, and precludes toxicity to normal cells [55]. The avidity of the nanocomplexes to the laminin receptor in the presence of the laminin receptor-specific Laminin-R antibody (H-2), suggested that cellular uptake was largely receptor-dependent, with both targeted nanocomplexes recording a marked reduction in uptake in the presence of the antibody. Although cationic NPs are expected to easily cross the cell membrane due to the negative membrane potential across the bilayer, the lower count observed for the T-Au-[PLL-g-PEG] NPs in spite of its cationic nature may be due to its larger size (147 nm compared to the 98 nm of T-Au-PEG).

The high negative membrane potential (−180 mV) perpetuated by oxidative phosphorylation restricts the movement of negatively charged particles into the inner mitochondrial membrane. In its hyperpolarized state, as in cancers, mitochondrial membrane potential may increase to as high as −210 mV, encouraging a high amplitude translocation of cationic molecules across the inner membrane. This is one of the factors responsible for the higher susceptibility of cancer mitochondria to mitochondriotropic agents compared to those of noncancer cells. T-Au-[PLL-g-PEG] due to its highly cationic nature, showed the highest mitochondrial localization compared to Au-[PLL-g-PEG], T-Au-PEG and Au-PEG in all three cell lines. Although, T-Au-PEG also demonstrated appreciable mitochondrial localization, its lower mitochondrial accumulation compared to T-Au-[PLL-g-PEG] in spite of a high cellular uptake, may have been influenced by its negative surface charge hampering its interaction with the hyperpolarized mitochondrial membrane. Studies on the relationship between NP size and mitochondrial uptake reported optimum uptake at a particle size between 80 and 100 nm, with a gradual decline till about 210 nm, after which uptake is negligible [47].

The observed tolerance of NPs and drug loaded nanocomplexes in the noncancer HEK293 cells, compared to the toxicity of the free BA affirms their biocompatibility and potential to prevent cargo-associated toxicity in normal cells. Their negligible impact on HEK293 cell viability may be associated with their high affinity for the laminin receptor, which is not overexpressed in these cells as in cancer cells, in addition to the normal functional state of the mitochondria in the HEK293 cells. In the cancer cells, drug-loaded nanocomplexes showed significant cytotoxicity compared to the free drug across all concentrations. T-Au-PEG-BA and T-Au[PLL-g-PEG]-BA displayed a concentration dependent inhibition of cell growth in all three cancer cell lines. Cancer cell mitochondria are dysfunctional and significantly hyperpolarized, enhancing the mitochondrial accumulation of cationic or mitochondrial targeted NPs and their cargo [47,56]. This explained their high susceptibility to T-Au-PEG-BA and T-Au[PLL-g-PEG]-BA treatments. Some studies have reported that negatively charged NPs do not readily translocate into the mitochondria due to their high negative membrane potential, while other studies have established the affinity of EGCG to the mitochondria [49,57]. A 90–95% accumulation of radioactive ^3^Au-EGCG in the mitochondria of primary cultures of rat cerebellar granule neurons was reported [58]. Hence, T-Au-PEG-BA in spite of its negative surface charge may have been influenced by the EGCG component of the nanocomplex. Overall, the lower impact of free BA on the viability of the cancer cells compared to that of the targeted nanocomplexes emphasizes the challenge of poor pharmacokinetics in chemotherapy, while demonstrating the importance of specialized nanodelivery platforms such as T-Au-PEG and T-Au[PLL-g-PEG] in mitigating this challenge.

Tumors are reliant on anerobic respiration for energy generation, and mitochondrial membrane hyperpolarization is a consequent anomaly arising from the accumulation of the proton motive force across the inner mitochondria membrane. The dissipation of this membrane potential compromises the mitochondrial membrane integrity, setting-off a cascade of events leading to cell death [59]. While its antiproliferative effect has been linked to a host of mechanisms, the impact of BA on the modulation of apoptotic signaling, the consequent opening of the mitochondria membrane permeability transition pore through ROS generation, and the release of cytochrome c has been established [13,60]. The assessment of the impact of free BA, and the nanocomplexes on mitochondrial membrane potential revealed highly significant membrane depolarizations by T-Au-PEG-BA and T-Au[PLL-g-PEG]-BA compared to the untargeted (Au-PEG-BA and Au-[PLL-g-PEG]-BA), and free BA, highlighting the efficacy of this targeted approach. Moreover, T-Au-[PLL-g-PEG]-BA showed higher depolarization of the cancer cells, compared to T-Au-PEG-BA. Considering the cytotoxicity initially recorded for T-Au-PEG-BA, and its low impact on the mitochondrial membrane potential, attests to the possibility of other cytoplasmic targets, and the induction of non-mitochondrial dependent auxiliary pathways of cell death, as reported by other investigators [60,61,62,63].

The cascade of events following the loss of mitochondrial membrane potential due to mitochondrial membrane disruption includes the release of cytochrome c from its intermembrane stores and the activation of initiator caspases, and the subsequent activation of executioner caspases such as caspases 3 and 7. A similar trend to that of the membrane potential analysis was noted, with targeted constructs eliciting the highest percentage of caspase 3/7-mediated cell death compared to both the untargeted and free BA treatments. Overall, T-Au-[PLL-g-PEG]-BA demonstrated a higher efficacy compared to T-Au-PEG-BA, corroborating earlier inference that the comparable cytotoxic effect of T-Au-PEG-BA is likely connected to its impact on other cytoplasmic targets as influenced by its higher cellular uptake and lower mitochondrial localization. Hence, the apparent effectiveness of T-Au-[PLL-g-PEG]-BA may be linked to its superior mitochondria targeting potential as demonstrated in the localization study. These findings further emphasize the potential of efficient mitochondrial targeting platforms, especially, T-Au-[PLL-g-PEG] to enhance drug pharmacokinetics. The significant apoptogenic potential of T-Au-[PLL-g-PEG]-BA was again confirmed by fluorescent apoptotic studies employing the AO/EB dual stain.

Studies have established a relationship between cell cycle arrest and caspase dependent apoptosis [64,65]. The upregulation of the p53 protein, its downstream effector, p21, and the downregulation of cyclins D and E, and an arrest at the G1 phase of the cell cycle are some of the consequent highlights of apoptosis induction [66]. The effect of the targeted nanocomplexes on the cell cycle revealed arrests at the G0/G1 phase compared to the control in all the cells, with T-Au-[PLL-g-PEG]-BA having a significant impact in the Caco-2 and MCF-7 cells, compared to the control, free BA and T-Au-PEG-BA. These findings are in consonance with earlier reports on the effect of BA-rich *Dillenia suffruticosa* root extracts in MCF-7 cells and as well as free BA in oral squamous cell cancer [67,68].

## 4. Materials and Methods

### 4.1. Materials

Tetrachloroaurate (III) trihydrate (HAuCl_4_-3H_2_O), poly-L-lysine hydrobromide (PLL, Mw 1000–4000), (−)-epigallocatechin gallate (EGCG), N-hydroxysuccinimide (NHS), betulinic acid, N-(3-dimethyl aminopropyl)-N′-ethyl carbodiimide hydrochloride (EDC), 4-(dimethylamino)pyridine (DMAP), (4-Carboxybutyl)triphenyl-phosphonium bromide, dialysis tubing (MWCO 2000) and the bicinchoninic acid (BCA) assay kit were obtained from Sigma-Aldrich, St. Louis, MO, USA. Polyethyleneglycol 2000 (Mw 1800), acridine orange, ethidium bromide, and 3-(4-,5-dimethylthiazol-2-yl)-2,5-diphenyltetrazolium bromide (MTT) were supplied by Merck, Darmstadt, Germany, while cell lysis buffer (5x) was purchased from Promega Corporation, Madison, USA. The Muse^®^ cell cycle, caspase and mitopotential kits were sourced from Luminex Corporation, Austin, TX, USA All other reagents were locally purchased and of analytical grade. The human embryonic kidney (HEK293), colorectal adenocarcinoma (Caco-2), breast adenocarcinoma (MCF-7), and cervical adenocarcinoma (HeLa) cell lines were initially procured from the American Type Culture Collection (ATCC), Manassas, VA, USA. Minimum essential medium (EMEM), trypsin-versene and penicillin/streptomycin (10,000 U/mL penicillin, 10,000 U/mL streptomycin) were obtained from Lonza Bio Whittaker, Verviers, Belgium. Fetal bovine serum (FBS) was supplied by Hyclone, GE Healthcare, Utah, USA. All sterile tissue culture grade plasticware were obtained from Corning Inc. (New York, NY, USA). Cell culture medium was supplemented with 10% FBS and 1% penicillin/streptomycin amphotericin B mixture, and incubated at 37 °C in 5% CO2.

### 4.2. Synthesis of EGCG Capped AuNPs

We synthesized EGCG-capped AuNPs by the reduction of hydrogen tetrachloroaurate (III) trihydrate (HAuCl_4_-3H2O) with EGCG as previously described [21,40]. Briefly, 100 µL of 0.3 mM HAuCl4-3H2O was added to a 10 mL stirring 0.11 mM solution of EGCG in water. The mixture was stirred for an additional 10 min to allow for complete AuNP formation. Thereafter, the suspension was purified by dialysis (MWCO 2000 Da) against ultrapure water over 12 h. The formation of EGCG capped AuNPs was confirmed by UV spectroscopy.

### 4.3. Synthesis of AuNP-BA

BA was loaded into the EGCG capped AuNPs by Steglich esterification involving the carboxylic group of BA and the hydroxyl groups of EGCG [69]. BA (0.5 mM) and 4-dimethylaminopyridine (0.4 mM) were dissolved in 2 mL deionized water and stirred for 1 h. The mixture was then added with stirring to the AuNP suspension in a 1:5 final ratio (*v*/*v*). Thereafter, EDC (0.134 g, 0.7 mM in 2 mL deionized water) was added dropwise to the mixture, which was stirred overnight at room temperature for the reaction to proceed. The AuNP-BAs were purified by dialysis as in Section 4.2.

### 4.4. Synthesis of Poly-L-Lysine-Graft-(g)-Polyethylene Glycol Copolymer (PLL-g-PEG)

PEG-imidazole (PEG-CI) was first synthesized. Approximately, 0.2 g of 1, 1′-carbonyl diimidazole (CDI) was dissolved in 10 mL dioxane and added to a 0.1 g dry PEG solution in 25 mL of toluene. The mixture was stirred overnight at 50 °C and concentrated by rotary evaporation. The resultant yellow liquid was diluted with 50 mL dichloromethane and transferred to an ice bath where 20 mL of 1 M NaCl was added while stirring until effervescence ceased. The organic layer was then separated from the cloudy aqueous layer, washed twice with water and dried for 12 h with anhydrous Na_2_SO_4_. The Na_2_SO_4_ was then filtered and the filtrate concentrated and lyophilized. 1H NMR (600 MHz, CDCl_3_) δ 8.88, 8.11, 7.49, 7.30, 7.29, 3.75, 3.74, 3.74, 3.68, 3.68, 3.67, 3.66, 3.66, 3.63, 3.63, 2.97 (Supplementary Materials Figure S1).

Poly-L-lysine (PLL, 5 mg) was dissolved in freshly prepared sodium borate buffer (pH 9), followed by the addition of 20 mg of PEG-CI (based on the 82% yield) and stirred overnight. The solution was then dialyzed as in Section 4.2, concentrated and lyophilized. ^1^H NMR (400 MHz, D_2_O) δ 4.70, 4.23, 4.14, 3.62, 3.01, 2.86, 1.81, 1.58, 1.29 (Supplementary Materials Figure S2).

### 4.5. Synthesis of Triphenylphosphine-PLL-g-PEG (T-PLL-g-PEG)

The synthesis of T-PLL-g-PEG was accomplished by amide coupling between the free carboxylic end of (4-carboxybutyl) triphenyl-phosphonium bromide (TPP–(CH_2_)_4_–COOH) and the free amino groups of PLL. TPP–(CH_2_)_4_–COOH (0.1 g, 0.226 mM), NHS (0.04 g, 0.339 mM) and EDC (0.0433 g, 0.226 mM) were dissolved in 5 mL of water and stirred for 1 h. To this was added PLL-g-PEG-OH (10 mg/mL) with stirring overnight at room temperature. The product was purified by dialysis as in Section 4.2 for 6 h and then concentrated and lyophilized. ^1^H NMR (400 MHz, DMSO) δ 7.90, 7.81, 7.76, 6.07, 6.00, 3.51, 2.99, 2.89, 2.59, 2.46, 2.39, 1.71, 1.61, 0.99 (Supplementary Materials Figure S3).

### 4.6. Synthesis of T-Au-[PLL-g-PEG]-BA), Au-PLL-g-PEG-BA, and T-Au-PEG-BA

Lyophilized T-(PLL-g-PEG) was resuspended in ultrapure water (2 mg/mL) and added dropwise to a stirring AuNP-BA suspension in a 1:10 (*v*/*v*) final ratio. The mixture was stirred for 6 h, and thereafter dialyzed overnight against water to remove any unreacted T-(PLL-g-PEG). This procedure was repeated for formation of untargeted constructs using PLL-g-PEG. T-Au-PEG-BA NPs were synthesized by a sequential conjugation of PEG-CI and TPP–(CH_2_)_4_–COOH to the OH groups on AuNP-BA. A PEG-CI solution (10 mg/mL) was added dropwise to a stirring AuNP-BA suspension (pH 9). The reaction was allowed to proceed overnight, and thereafter dialyzed, as previously for 8 h to remove unreacted PEG-CI. Thereafter, TPP–(CH_2_)_4_–COOH (0.29 mM) and 4-dimethylaminopyridine (0.29 mM) were dissolved in 2 mL deionized water and stirred for 1 h. The mixture was then added to the AuNP-PEG-BA suspension in a 1:10 final ratio (*v*/*v*) with stirring. Thereafter, EDC (0.29 mM in 1 mL deionized water) was added dropwise to the stirring mixture. The reaction was allowed to proceed overnight at room temperature and thereafter, the product was purified by dialysis as in Section 4.2.

### 4.7. Drug Loading Efficiency

A 2 mL aliquot of the NP–drug complex (nanocomplex) was degraded by using 1.0 mM potassium iodide, and then centrifuged at 12,000× *g* for 10 min to obtain a clear supernatant. The supernatant was subjected to UV-vis spectroscopy at 210 nm, while the corresponding BA concentration was deduced from a standard curve. The procedure was repeated 5 times, and loading efficiency determined from the average by the Equation below:

Loading efficiency (%) = (Encapsulated drug)/(Total drug added) × 100(1)

### 4.8. Nanoparticle Characterization

Changes in optical property and stability of the AuNPs upon synthesis and functionalization were monitored via UV-vis spectroscopy (Jasco V-730 Bio Spectrophotometer, JASCO Corporation, Hachioji City, Japan) over a wavelength range of 400–800 nm. Bond formation and functionalization of the AuNPs were determined by changes in absorption bands as evidenced by Fourier Transform Infra-red (FTIR) spectrometry (ATR-FTIR spectroscopy PerkinElmer, Inc. USA). NP shape, size and dispersity were determined from images generated by transmission electron microscopy (TEM) (JEM 1010, JEOL, Tokyo, Japan). Hydrodynamic diameters, zeta-potential and stability were measured by nanoparticle tracking analysis (NTA) (Nanosight NS500, Malvern, Worcestershire, UK), at 25 °C, and analyzed using the NTA version 3.2 software.

### 4.9. Cell Culture

All cell culture protocols were conducted under sterile conditions in an Airvolution Class II biosafety laminar flow hood (United Scientific, South Africa). Human colorectal adenocarcinoma (Caco-2), cervical carcinoma (HeLa), breast adenocarcinoma (MCF-7) and embryonic kidney (HEK293) cells were grown as adherent cultures at 37 °C under 5% CO_2_ (Steri-Cult CO_2_ incubator with class 100 HEPA filtration, Thermo Electron Corporation), in 25 cm^2^ culture flasks containing 5 mL of complete medium (EMEM supplemented with FBS 10% (*v*/*v*) and antibiotic (100 U/mL penicillin, 100 µg/mL streptomycin, 0.25 µg/mL amphotericin B)). Medium was routinely renewed until cells reached confluency. Confluent cells were trypsinized and cryopreserved, or propagated to increase cells numbers, or seeded into multiwell plates for cell-based experiments. All cell-based experiments were conducted in triplicate.

### 4.10. Cellular Uptake Studies

Cellular uptake was undertaken in HeLa cells that were seeded in 48-well plates (2.0 × 10^3^ cells/well) and incubated for 12 h. Cells in selected wells were incubated with the Laminin R antibody (H-2) (5 µg/mL) for 1 h prior to medium replacement. Targeted NPs (T-Au-[PLL-g-PEG] and T-Au-PEG) were then added at 80 µg/mL and cells incubated for 6 h. Thereafter, cells were washed with cold PBS to remove residual medium and NPs. Cells were then trypsinized, lysed and centrifuged at 3000× *g* for 5 min to remove all cell debris. The resulting supernatant was then filtered through a 30-kDa MWCO Amicon^®^ filter (Millipore) to remove other cellular contaminants. The filtrate was analyzed by NTA to determine the constituent nanoparticle concentration. Untreated cells were subjected to the same procedure and served as the control.

### 4.11. Quantitative Determination of Nanoparticle Distribution by ICP-OES Analysis

Caco-2, HeLa and MCF-7 cells (2.0 × 10^5^/mL) were seeded into 75 cm^2^ flasks, containing 25 mL EMEM and incubated overnight at 37 °C in 5% CO_2_. Following medium replacement, 1.58 mg/mL of targeted and untargeted NPs were added, and cells incubated for 12 h at 37 °C in 5% CO_2_. The medium was then discarded, and the cells washed 3 times with cold PBS (pH 7.4.) Cells were harvested by trypsinization and subsequent centrifugation at 300× *g* for 5 min at 4 °C. The cell pellet was resuspended in 50 µL of 10% cell lysis buffer (1×) and placed on ice for 5 min, with vortexing at 1 min intervals. Thereafter, 450 µL of a 0.25 M sucrose buffer (pH 7.4) was added and the cell suspension centrifuged twice at 700× *g* at 4 °C to remove cellular debris. The supernatant was further centrifuged at 12,000× *g* for 10 min at 4 °C, to obtain the mitochondrial fraction (pellet), and cytoplasmic fraction (supernatant). The mitochondrial fraction was washed once in sucrose buffer at 10,000× *g* for 10 min at 4 °C. Both samples were digested in aqua regia (2 h at 90 °C) and subjected to inductively coupled plasma–optical emission spectrometry (ICP-OES) analysis for the determination of elemental gold concentration. ICP-OES was conducted on a Perkin Elmer Optima 5300 DV Optical Emission Spectrometer, and calibrated using 0, 50, 100, 200, 300, 400 and 500 parts per billion (ppb) of Au standard stock solution. Results were expressed in ng/µg protein. The analysis was conducted in triplicate.

### 4.12. MTT Assay

The MTT cytotoxicity assay determines the percentage viability of a cell population as a function of the ability of live cells to reduce the MTT ((3-(4,5-dimethylthiazol-2-yl)-2,5-diphenyltetrazolium bromide) to formazan by the NAD(P)H dependent oxido-reductase enzyme system [70]. Confluent HEK293, Caco-2, HeLa, and MCF-7 cells were seeded (2.0 × 10^3^ cells/well) into 96-well plates and incubated overnight at 37 °C in 5% CO_2_. The medium was then replaced, and NPs at varying concentrations were added, followed by incubation at 37 °C in 5% CO_2_ for 48 h. At the end of the incubation period, the spent medium was replaced with fresh medium (100 µL) containing 10 µL (5 mg/mL in PBS) MTT reagent, and cells incubated as above for 4 h. Control wells containing untreated cells were treated similarly. Thereafter, the MTT/medium was removed and 100 µL DMSO was added to solubilize the formazan product. Absorbance was read against a DMSO blank at 540 nm using a Mindray MR-96A microplate reader (Vacutec, Hamburg, Germany). Cell viability was determined using the Equation below:

Cell Viability (%) = (OD _treated cells)_/(OD _control cells_) × 100 (2)

### 4.13. Cell Cycle Analysis

Caco-2, HeLa, and MCF-7 cells were seeded (4.0 × 10^3^ cells/well) into 48-well plates and incubated for 12 h (37 °C and 5% CO_2_). After medium replacement, cells were treated with both targeted and untargeted nanocomplexes, and free BA at the equimolar BA concentration of 25 µM, and incubated for 48 h. The cells were then rinsed twice with cold PBS (pH 7.4), trypsinized and centrifuged at 300× *g* for 5 min. Cells were then washed once with PBS, suspended in 70% cold ethanol, and then incubated for 3 h at −20 °C. Ethanol was removed by centrifugation (300× *g* for 5 min) and cells washed once with PBS. Thereafter, 200 µL of the Muse^®^ cell cycle reagent (containing premixed propidium iodide and RNase A), was added to each tube, and incubated for 30 min in the dark, at room temperature. Thereafter, cells were assessed using the Muse™ cell analyzer (Luminex Corporation, Austin, TX, USA).

### 4.14. Mitopotential Assay

Cells were seeded and treated as in Section 4.12. After a 48 h incubation, cells were trypsinized, centrifuged at 300× *g* for 5 min, washed once with PBS and resuspended in 100 µL PBS. Approximately, 95 µL Muse^®^ mitopotential working solution was added to each tube, and cells incubated at 37 °C for 20 min. Thereafter, 5 µL of 7-aminoactinomycin D (7-AAD) was added, followed by incubation at room temperature for 5 min. Mitochondrial membrane potential was assessed using the Muse™ cell analyzer.

### 4.15. Caspase 3/7 Analysis

Cells were seeded and treated as in Section 4.12. After incubation, cells were trypsinized, centrifuged and suspended in PBS. To a 50 µL cell suspension, 5 µL caspase-3/7 working reagent was added, and cells incubated at 37 °C for 30 min. Thereafter, 150 µL 7-AAD working solution was added to each tube, thoroughly mixed, and analyzed using the Muse™ cell analyzer.

### 4.16. Apoptosis Assay

A qualitative assessment of apoptosis induction in the selected normal and cancer cells was conducted by the acridine orange/ethidium bromide (AO/EB) dual staining method [28]. Cells were seeded as in Section 4.12 and incubated for 24 h at 37 °C in 5% CO_2_. Subsequently, the spent medium was replaced, and cells incubated for a further 48 h in the presence of the nanocomplexes and the free drug at their pre-determined IC_50_ concentrations. Untreated cells were used as controls. After the incubation the cells were washed twice with cold PBS, 15 µL of the AO/EB dye mixture (0.1 mg/mL:0.1 mg/mL) was added, and cells incubated for 5 min at ambient temperature. The cells were then washed with PBS to remove any unabsorbed dye and viewed under an Olympus fluorescence microscope (Wirsam Scientific and Precision Eq. LTD., Johannesburg, South Africa). Images were captured using a CC12 fluorescence camera and Analysis Five Software (Olympus Soft Imaging Solutions, Olympus, Japan) at 200× magnification.

### 4.17. Statistical Analysis

Data are presented as mean ± standard deviation (SD; *n* = 3). Data were analyzed using GraphPad Prism Version 7.3 (GraphPad Software Inc., San Diego, CA, USA). A two-way ANOVA with a post-hoc Tukey test was used to identify significant differences among the groups, while differences between two values were performed using an unpaired Student’s *t* test. Differences were considered statistically significant at * *p* < 0.05. All experiments were conducted in triplicate.

## 5. Conclusions

This study highlights the merit of NP application in drug delivery as a tool for improving drug bioavailability and pharmacokinetics. Furthermore, it provides a proof of concept for the subcellular delivery of therapeutics as an alternative approach to improving selectivity. As demonstrated in this study, the targeted T-Au-[PLL-g-PEG]-BA and T-Au-PEG-BA showed measurable impact at lower doses with negligible side effects in normal cells. They effected significant cytotoxicity in the Caco-2, HeLa and MCF-7 cell lines, compared to the free drug. The underlying mechanism involved the mitochondrial dependent pathway of apoptosis, with T-Au-[PLL-g-PEG]-BA being the most efficient of the nanocomplexes. This study also confirmed the laminin receptor-dependent uptake of EGCG-capped NPs and demonstrated their potential suitability as a platform for mitochondrial targeted delivery of therapeutics, following functionalization. Importantly, we established the proficiency of the T-Au-[PLL-g-PEG] nano-construct as a favorable platform for mitochondrial delivery of BA, with attention to its enabling physicochemical properties. Further research is however required, especially in vivo studies, to assess its potential for clinical applications.

## Figures and Tables

**Table 1:** Hydrodynamic size, zeta potential (ζ), polydispersity indices (PDI) and drug loading efficiencies of nanoparticles.

NPs	Hydrodynamic Size (nm)	PDI	ζ Potential (mV)	BA Loading Efficiency (%)
EGCG-AuNP	127.3 ± 3.1	0.10	−28.3 ± 1.6	-
Au-PEG-BA	110.1 ± 5.3	0.22	−25.0 ± 0.9	25.4 ± 0.04
T-Au-PEG-BA	97.1 ± 2.5	0.035	−23.1 ± 1.1	25.4 ± 0.12
Au-[PLL-g-PEG]-BA	147.2 ± 4.7	0.13	+11.8 ± 1.6	21.0 ± 1.2
T-Au-[PLL-g-PEG]-BA	119.2 ± 3.5	0.11	+23.4 ± 0.5	21.0 ± 0.7

**Table 2:** Estimated IC_50_ values of BA, targeted and untargeted nanocomplexes.

	Au-PEG-BA	T-Au-PEG-BA	Au-[PLL-g-PEG]-BA	T-Au-[PLL-g-PEG]-BA	BA
	Estimated IC_50_ Values (µM)				
**Caco-2**	8.20	3.13	5.72	3.12	9.74
**HeLa**	25.37	6.51	23.64	3.26	17.73
**MCF-7**	53.74	13.2	22.25	13.13	36.31

## Data Availability

The data and contributions presented in the study are included in the article and Supplementary Materials. Further inquiries can be directed to the corresponding author.

## Supplementary Materials

The following are available online at https://www.mdpi.com/article/10.3390/ijms22105072/s1, Figure S1: ^1^H NMR of PEG-CI in CDCl3; Figure S2: ^1^H NMR PLL-g-PEG in D2O; Figure S3: ^1^H NMR PLL-g-PEG-TPP+ in DMSO.
