# Supplementary material for: Modified Gold Nanoparticles for Efficient Delivery of Betulinic Acid to Cancer Cell Mitochondria
Source: Int J Mol Sci. 2021 May 11;22(10):5072. doi: 10.3390/ijms22105072 (PMC8150271; doi:10.3390/ijms22105072)
Supplement: Supplementary file 1 [file ijms-22-05072-s001.zip › ijms-1191525-supplementary.pdf]

International Journal of *Molecular Sciences*

MDPI

Modified Gold Nanoparticles for efficient Delivery of Betulinic Acid to Cancer Cell Mitochondria

Olakunle Oladimeji[1], Jude Akinnyelu[1], Aliscia Daniels[1], Moganavelli Singh[1*]

[1]Nano-Gene and Drug Delivery Group, Discipline of Biochemistry, School of Life Sciences, University of KwaZulu-Natal, PrivateBag X54001, Durban, South Africa

***Correspondence:** Moganavelli Singh singhm1@ukzn.ac.za

Supplementary Material

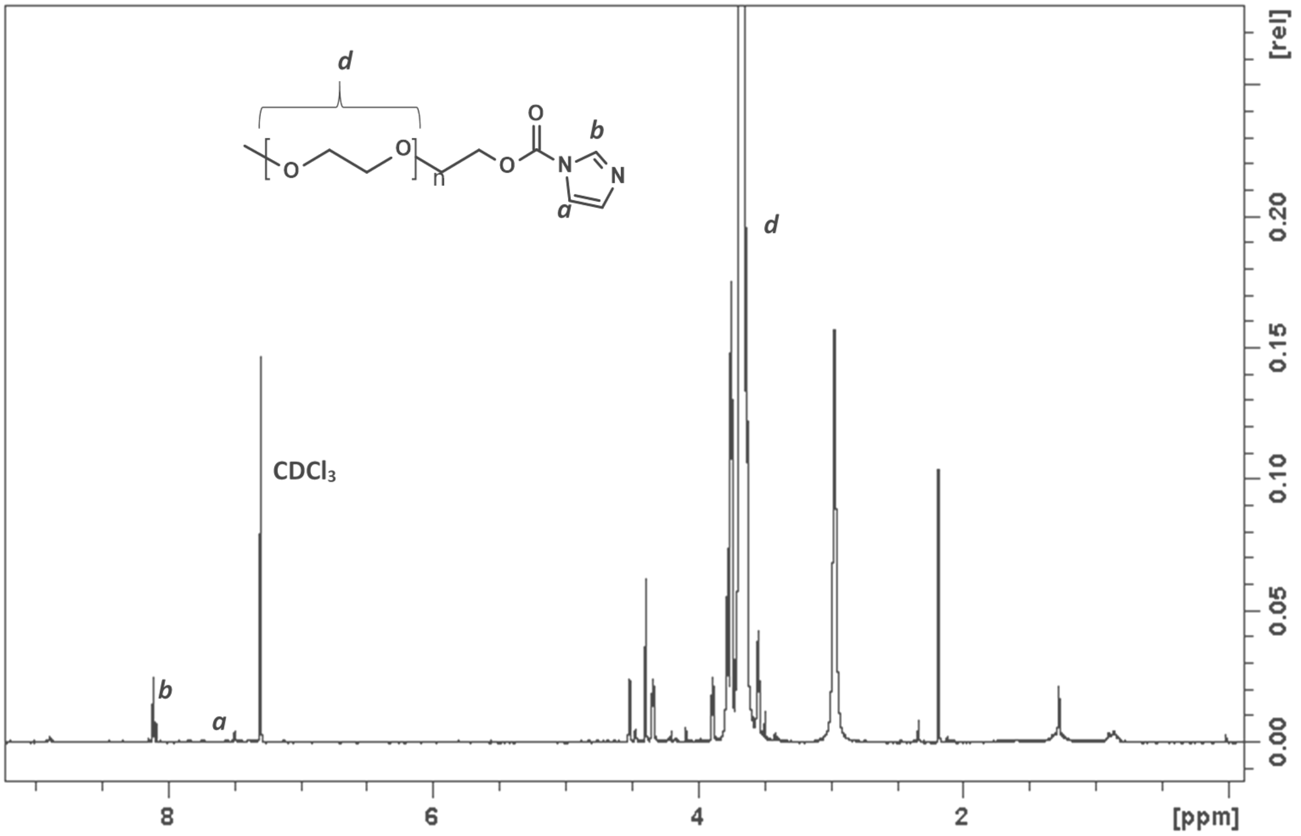

Supplementary Figure S1. ^{1}H NMR of PEG-CI in $CDCl_3$

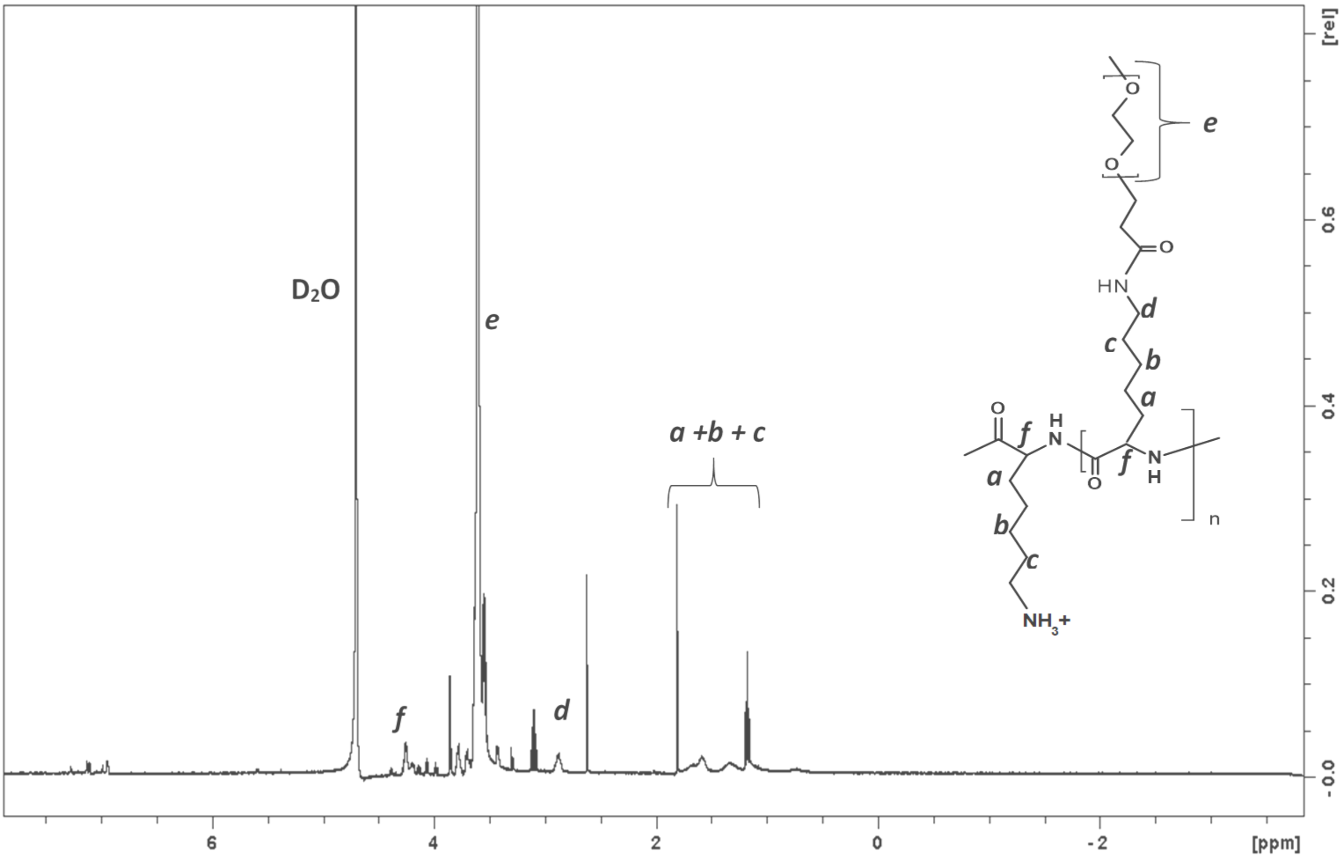

Supplementary Figure S2. ^1H NMR PLL-g-PEG in D_2O.

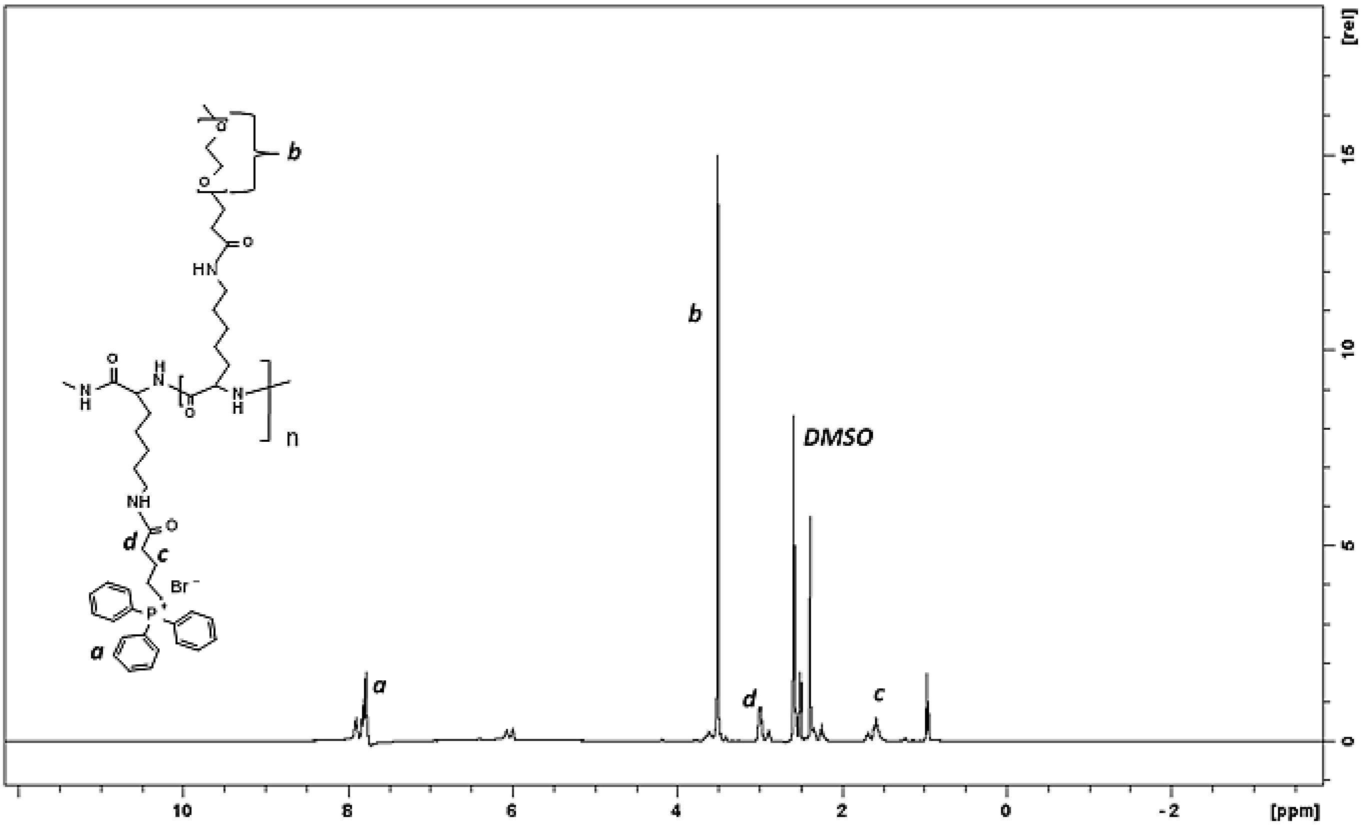

Supplementary Figure S3. ^{1}H NMR PLL-g-PEG-TPP+ in DMSO.
